# P-205. Awareness and Preferences in PrEP Services among Men Who Have Sex With Men in the Philippines: A Discrete Choice Experiment

**DOI:** 10.1093/ofid/ofaf695.427

**Published:** 2026-01-11

**Authors:** Rodenie Olete, Patrick Eustaquio, Warittha Tieosapjaroen, Kate Leyritana, Jason J Ong, Heather-Marie A Schmidt, Nittaya Phanuphak, Curtis Chan, Benjamin Bavinton

**Affiliations:** Department of Public Health, National Cheng Kung University, Tainan City, Tainan, Taiwan; Independent Researcher, Metro Manila, Philipines, Pasig City, National Capital Region, Philippines; Monash University, Melbourne, Victoria, Australia; Sustained Health Initiatives of the Philippines, Inc., Mandaluyong City, National Capital Region, Philippines; Monash University, Melbourne, Victoria, Australia; Joint United Nations Programme on HIV/AIDS, Geneva, Geneve, Switzerland; Institute of HIV Research and Innovation – Pribta Tangerine Clinic, Bangkok, Krung Thep, Thailand; Kirby Institute, University of New South Wales, Sydney, New South Wales, Australia; Kirby Institute, University of New South Wales, Sydney, New South Wales, Australia

## Abstract

**Background:**

Between 2010 and 2023, the Philippines experienced a 550% increase in HIV cases, with men who have sex with men (MSM) disproportionately affected. Although the national rollout of pre-exposure prophylaxis (PrEP) began in 2021, uptake remains minimal. Structural and individual-level barriers, including cost, stigma, and limited service options, continue to hinder PrEP uptake. This study investigates Filipino MSM’s preferences for PrEP service attributes to guide person-centered HIV prevention strategies.

**Figure:**
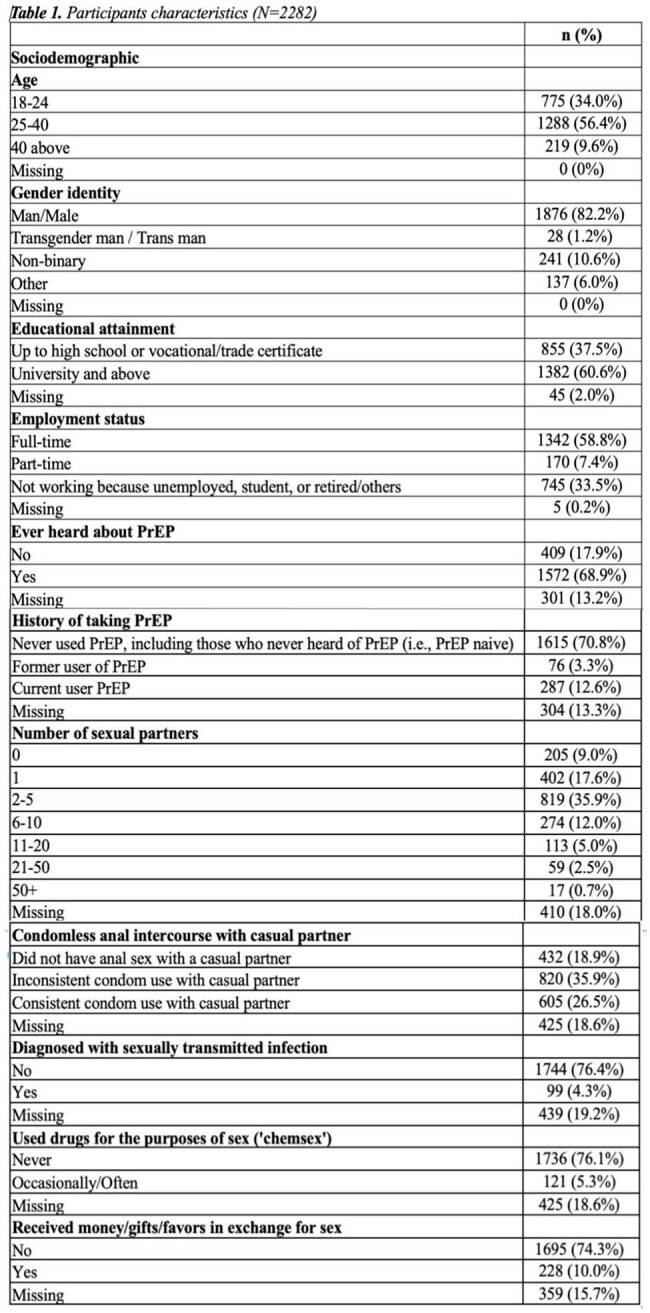

**Figure:**
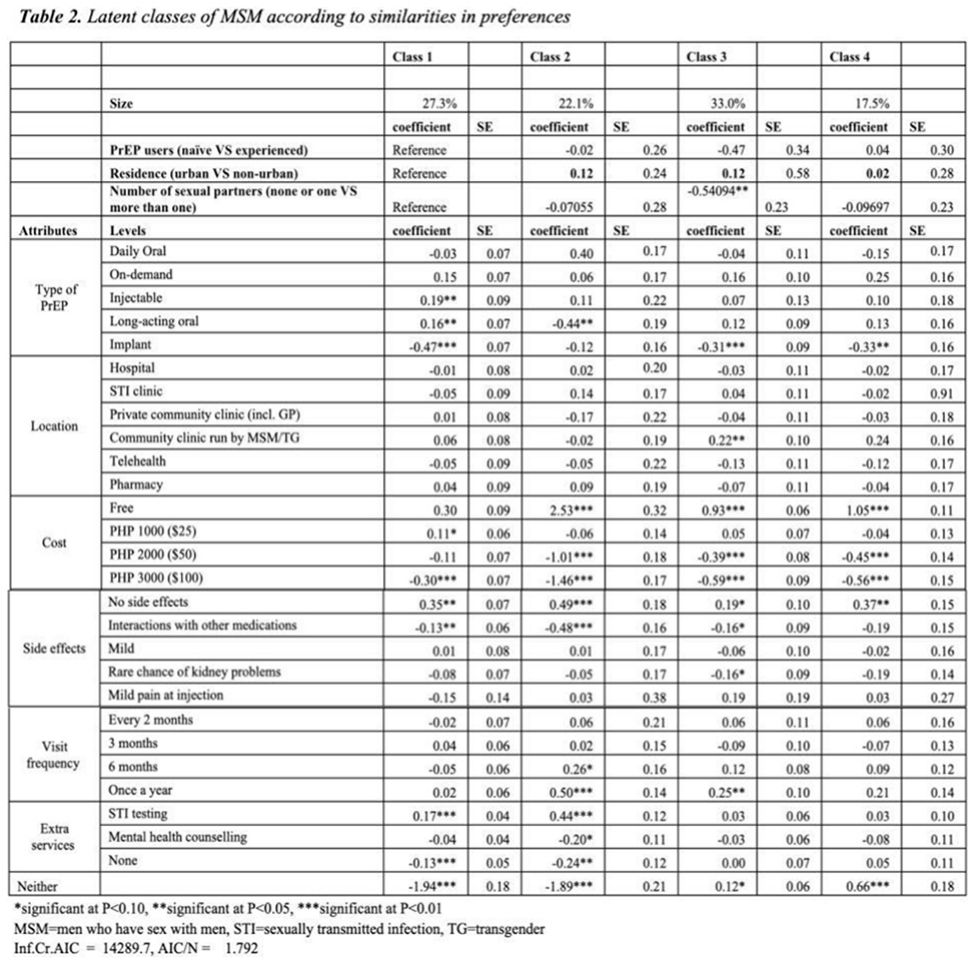

**Methods:**

We analyzed the Philippines data from PrEP APPEAL, a multi-country cross-sectional online survey conducted from May to November 2022. The survey included a discrete choice experiment (DCE) evaluating preferences for six PrEP service attributes: type (e.g. oral, long-acting injectable), service location, cost, side effects, visit frequency, and inclusion of additional services. We analyzed preferences using random parameters Logit and latent class models.

**Figure:**
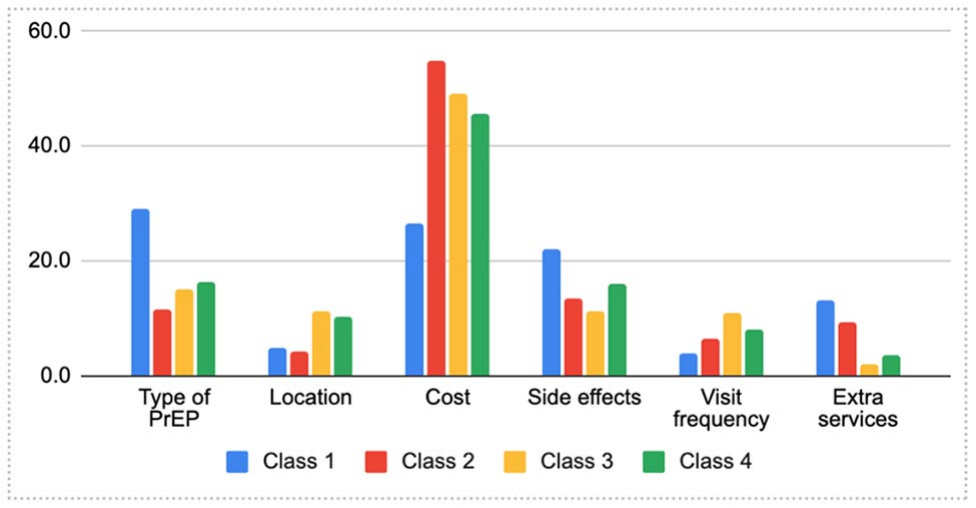

**Results:**

Among 2,282 MSM respondents (mean age: 28.8 years, SD: 7.9), 68.9% had heard of PrEP, but only 18.3% were current users (Table 1). Cost, formulation, and side effects most strongly influenced decision-making (Figure 1). Specifically, MSM preferred free oral PrEP accessed through community-led clinics, with minimal side effects and 6-12 month visits. Latent class analysis revealed four distinct subgroups with varying preferences: (1) “Long-Acting Preventive Realists” (27.3%), who prefer long-acting PrEP options without side-effects and with STI testing, (2) “Cost-Conscious Annually-Visiting Pragmatists” (22.1%), who prefer free daily oral PrEP without side effects and with annual visits for refill and STI testing, (3) “Community-based Organization (CBO)-Trusting Lowkey Clinic Visitors” (33.0%), who prefer annual visits at CBOs with low costs, and (4) “Easy-Going PrEP-Hesitants” (17.5), who disliked implants but otherwise no preference on other PrEP types and prefer free and without side-effects (Table 2).

**Conclusion:**

Findings underscore that a one-size-fits-all model is insufficient. National strategies must endorse a variety of community-led PrEP delivery models tailored to as many Filipino MSM with diverse preferences for PrEP products, cost, and visit frequency as possible.

**Disclosures:**

Jason J. Ong, PhD, MMed, MBBS, Australian National Health and Medical Research Council: Grant/Research Support|Gilead Sciences: Grant/Research Support Nittaya Phanuphak, MD, PhD, Aspire Scientific: Medical writing support|Gilead Sciences, Inc.: Grant/Research Support|Gilead Sciences, Inc.: Medical writing support|ViiV Healthcare: Grant/Research Support Benjamin Bavinton, PhD, Gilead Sciences: Grant/Research Support|Gilead Sciences: Honoraria|ViiV Healthcare: Grant/Research Support|ViiV Healthcare: Honoraria

